# Facilitating Reparative Dentin Formation Using Apigenin Local Delivery in the Exposed Pulp Cavity

**DOI:** 10.3389/fphys.2021.773878

**Published:** 2021-12-10

**Authors:** Yam Prasad Aryal, Chang-Yeol Yeon, Tae-Young Kim, Eui-Seon Lee, Shijin Sung, Elina Pokharel, Ji-Youn Kim, So-Young Choi, Hitoshi Yamamoto, Wern-Joo Sohn, Youngkyun Lee, Seo-Young An, Chang-Hyeon An, Jae-Kwang Jung, Jung-Hong Ha, Jae-Young Kim

**Affiliations:** ^1^Department of Biochemistry, School of Dentistry, IHBR, Kyungpook National University, Daegu, South Korea; ^2^Department of Dental Hygiene, College of Health Science, Gachon University, Incheon, South Korea; ^3^Department of Oral and Maxillofacial Surgery, School of Dentistry, IHBR, Kyungpook National University, Daegu, South Korea; ^4^Department of Histology and Developmental Biology, Tokyo Dental College, Tokyo, Japan; ^5^Pre-major of Cosmetics and Pharmaceutics, Daegu Haany University, Gyeongsan, South Korea; ^6^Department of Oral and Maxillofacial Radiology, School of Dentistry, IHBR, Kyungpook National University, Daegu, South Korea; ^7^Department of Oral Medicine, School of Dentistry, IHBR, Kyungpook National University, Daegu, South Korea; ^8^Department of Conservative Dentistry, School of Dentistry, IHBR, Kyungpook National University, Daegu, South Korea

**Keywords:** inflammation, osteodentin, pulp cavity, reparative dentin formation, signaling modulation

## Abstract

Apigenin, a natural product belonging to the flavone class, affects various cell physiologies, such as cell signaling, inflammation, proliferation, migration, and protease production. In this study, apigenin was applied to mouse molar pulp after mechanically pulpal exposure to examine the detailed function of apigenin in regulating pulpal inflammation and tertiary dentin formation. *In vitro* cell cultivation using human dental pulp stem cells (hDPSCs) and *in vivo* mice model experiments were employed to examine the effect of apigenin in the pulp and dentin regeneration. *In vitro* cultivation of hDPSCs with apigenin treatment upregulated bone morphogenetic protein (BMP)- and osteogenesis-related signaling molecules such as BMP2, BMP4, BMP7, bone sialoprotein (BSP), runt-related transcription factor 2 (RUNX2), and osteocalcin (OCN) after 14 days. After apigenin local delivery in the mice pulpal cavity, histology and cellular physiology, such as the modulation of inflammation and differentiation, were examined using histology and immunostainings. Apigenin-treated specimens showed period-altered immunolocalization patterns of tumor necrosis factor (TNF)-α, myeloperoxidase (MPO), NESTIN, and transforming growth factor (TGF)-β1 at 3 and 5 days. Moreover, the apigenin-treated group showed a facilitated dentin-bridge formation with few irregular tubules after 42 days from pulpal cavity preparation. Micro-CT images confirmed obvious dentin-bridge structures in the apigenin-treated specimens compared with the control. Apigenin facilitated the reparative dentin formation through the modulation of inflammation and the activation of signaling regulations. Therefore, apigenin would be a potential therapeutic agent for regenerating dentin in exposed pulp caused by dental caries and traumatic injury.

## Introduction

Reparative dentinogenesis is the biological regeneration of dentin from new odontoblast-like cells when a dental injury is severe and reaches up to the dental pulp (Smith et al., 1995; Simon et al., 2012). A range of signaling molecules such as transcription, autocrine, and paracrine factors plays significant roles in dentin secretion and regeneration (Angelova Volponi et al., 2018; Baranova et al., 2020). Unlike enamel, dentin would be regenerated and modified by mature odontoblasts (Simon et al., 2012). During dental caries and lesions, dental pulp cells trigger a repair response to secrete dentin matrix to prevent spreading infection (Shah et al., 2020); and depending on the degree of severity, the secreted dentin is either tubular or osteodentin during reactionary and reparative dentinogenesis (Ricucci et al., 2014).

The restoration of the damaged pulpal tissue and the destroyed tooth structure has been still a challenge in the endodontic field. Recent studies are focused on using different growth factors and related signaling molecules before designing new drugs and biocapping materials (Bottino et al., 2017; Moussa and Aparicio, 2019). Previous reports highlighted different approaches such as mineral calcium phosphates, bioactive extracellular matrix, and stem cells for pulp healing and dentin regeneration (Goldberg et al., 2009; Lacerda-Pinheiro et al., 2012; Dimitrova-Nakov et al., 2014; Njeh et al., 2016). Particularly, most of the reports showed the effects of capping molecules in the reparative dentin formation (Accorinte et al., 2008; Huang, 2011; Smith et al., 2012; Tran et al., 2019). Besides these, (1) the protease inhibitor, bortezomib (Jung et al., 2017), (2) the transforming growth factor-β (TGF-β) family ligands, such as bone morphogenetic proteins (BMPs; Kwak and Lee, 2019), (3) glycogen synthase kinase (GSK-3) antagonists (Neves et al., 2017), (4) bone sialoprotein (BSP; Decup et al., 2000), (5) dentin phosphophoryn (DPP)/collagen composite (Koike et al., 2014), and (6) osteogenic protein-1 (OP-1), calcium hydroxide, and N-acetyl cysteine (NAC; Goldberg et al., 2001) have been incorporated in biomaterial scaffolds for dentin regeneration. For proper healing and regeneration of dentin, it is required to involve various signaling molecules and pathways. Specifically, previous reports emphasized the importance of Wnt and TGF-β signaling in the development of dental pulp and odontoblasts and the reparative dentin formation (Lim et al., 2014; Hunter et al., 2015; Jung et al., 2017; Niwa et al., 2018; Ali et al., 2019). Copious reports on detailed signaling molecules in dentin regeneration were prepared (Huang, 2011; Chmilewsky et al., 2014; Hunter et al., 2015; Karakida et al., 2019; Neves et al., 2020); however, it is still difficult to regenerate the dentin in the clinical field. These problems would result from the less focused study on controlling the inflammation in the early stage of tissue injury. During dentin-pulp regeneration, inflammation is the biggest hurdle for proper regeneration of pulp-dentin complex along with dental pulp tissue proliferation and remodeling (Shah et al., 2020). Inflammatory and immunological aspects of dental pulp repair should be taken into consideration during cavity treatment (Smith, 2002; Goldberg et al., 2008). The widely accepted paradigm both in the pulp and other bodily sites is that healing can only occur after removal of the infection, enabling a significant dampening of inflammation (Duncan and Cooper, 2020; Neves et al., 2020). Therefore, drugs that have both anti-inflammatory properties showing low cytokine stimulation levels and facilitating tissue regeneration can be considered as the supplementary agent to facilitate the reparative dentine formation (Julier et al., 2017).

Apigenin (4′,5,7-trihydroxyflavone), a natural flavonoid found in fruits and vegetables, has the effective anti-inflammatory, antioxidant, and anticancer properties (Shukla and Gupta, 2010; Ginwala et al., 2019; Zhou et al., 2019; Javed et al., 2021). The pharmacological value of apigenin now draws attention to be applied in different research fields, such as cardiology, neurology, and immunology (Shukla and Gupta, 2010; Li et al., 2017; Jiao et al., 2019). Several experiments reported apigenin in inflammation and disease control by modulating different signaling cascades (Shukla and Gupta, 2010; Ozbey et al., 2019); however, research involving apigenin in dental hard tissue regeneration, especially dentin regeneration, is lacking. Therefore, we evaluated the roles of apigenin in dentin-bridge formation and regeneration, with local delivery of apigenin after pulpal cavity access preparation in mice molar. In addition, we examined the signaling regulations and cellular physiology modulated by apigenin during human dental pulp stem cell (hDPSC) differentiation.

## Materials and Methods

### Human Dental Pulp Stem Cells and Apigenin Treatment

**FIGURE 1:**
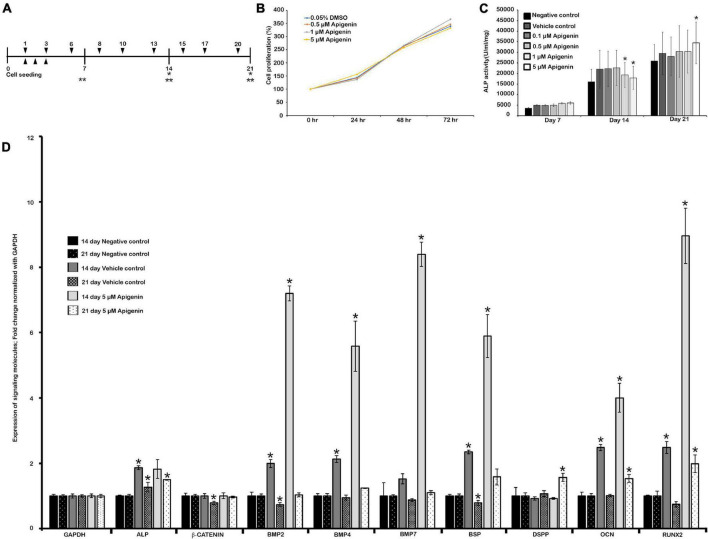
Human dental pulp stem cells (hDPSCs) and apigenin treatment. Experimental design showing hDPSCs cultured with apigenin for 3 weeks **(A)**; MTS assay showing cell viability and proliferation **(B)**; the effect of apigenin on ALP activity in hDPSCs **(C)**; altered expression patterns of signaling molecules after 5 μM apigenin treatment on hDPSCs at days 14 and 21 **(D)**. The highest ALP activity is observed following treatment with 5 μM apigenin at day 21. Bone morphogenetic protein (BMP)-related and osteogenesis-related signaling molecules are significantly upregulated in the apigenin-treated hDPSCs after 2 weeks. ▼ indicates the change of osteogenic medium, ▲ indicates MTS assay, * indicates real-time quantitative PCR (RT-qPCR), ** indicates ALP Assay **(A)**. * indicates *p* < 0.05 **(C,D)**.

The hDPSCs (cat# PT-5025, Lonza Bioscience, Basel, Switzerland) were cultured in DPSC SingleQuot Growth Medium (cat# PT-4516, Lonza Bioscience, Basel, Switzerland). For osteogenic differentiation, cells were seeded in collagen-coated 48-well plates at a density of 2 × 10^4^ cells/well. After 24 h of seeding, the medium was changed with osteogenic medium [a-MEM, 2% fetal bovine serum (FBS), 10 mM b-glycerophosphate, 50 μg/ml ascorbic acid, 100 nM dexamethasone] with or without apigenin (cat# 520-36-5, Sigma Aldrich, Saint Louis, MO, United States). The hDPSCs were cultured with three different sets: osteogenic medium only (negative control), osteogenic medium with 0.05% dimethyl sulfoxide (DMSO; vehicle control), and osteogenic medium with various concentrations of apigenin (experimental). The culture medium was changed three times per week (Figure 1A).

### Cell Viability Assay

Cell viability was evaluated by CellTiter 96^®^ AQueous One Solution Cell Proliferation Assay (MTS) (cat# G3582, Promega, Madison, WI, United States) in accordance with the instruction of the manufacturer. In brief, hDPSCs were seeded in 96-well plates at a density of 1 × 10^4^ cells/well with serum-free media, and apigenin treatments were performed 24 h after seeding. The MTS solution was added after 24, 48, and 72 h of apigenin treatment and incubated for 1 h at 37°C. Colorimetric changes were measured at 490 nm using a SpectraMax ABS Microplate Reader, Molecular devices, CA, United States.

### Alkaline Phosphatase Activity Assay

The effect of apigenin on alkaline phosphatase (ALP) activity in an osteoinductive environment was determined using ALP Assay Kit (cat # K412-500, BioVision, Milpitas, CA, United States). hDPSCs cultured in osteogenic medium for 7, 14, and 21 days were harvested and lysed with assay buffer. Then, the supernatant was treated with para-nitrophenyl phosphate (pNPP) solution for 1 h at room temperature. Finally, the reaction was terminated by adding a stop solution, and the absorbance was measured at 405 nm using a SpectraMax ABS Microplate Reader. Standard curve determination and ALP activity evaluations were performed according to the instructions of the manufacturer.

### Animals

For the pulp cavity preparation, 8-week-old male Institute of Cancer Research (ICR) mice were used. At least 10 mice were euthanized for 3, 5, and 42 days after cavity preparation to be used as control (*N* = 10) and experimental (*N* = 10) groups. Adult mice were housed under the following conditions: 22 ± 2°C, 55 ± 5% humidity, and artificial illumination lit between 5 a.m. and 5 p.m. with free access to food and water. All experimental protocols involving animal care and handling were approved by the Kyungpook National University, School of Dentistry, Animal Care and Use Committee (KNU-2015-136).

### Pulp Cavity Exposure and Drug Local Delivery

Mice were anesthetized by injecting Avertin (cat# T48402-5G, Sigma-Aldrich, MA, United States) intraperitoneally. The upper right first molar was ground using 0.6-mm round burr with a water spray immediately before pulp exposure. Subsequent pulpal exposure was conducted with ISO #15 K-file having 0.15 mm diameter (M-access, Dentsply Sirona, Ballaigues, Switzerland) to minimize heat stress under a dissecting microscope (S6, Leica, Wetzlar, Germany) as described previously (Jung et al., 2017). After that, 50 μM apigenin (experimental) or 0.05% DMSO (control) with Pluronic F-127 were delivered through the Hamilton syringe into the pulp cavity. After treatment, the teeth with exposed pulp were doubly sealed with Dycal (Dentsply Caulk, Milford, DE, United States) and light-cured composite resin (Jung et al., 2017). At least 10 mice (*N* = 10) were used for each group.

### Histology and Immunohistochemistry

The mice were sacrificed after 3, 5, and 42 days from local drug treatment, for which we have set up the experimental protocols in our laboratory (Jung et al., 2017). The sacrificed mice were fixed in 4% paraformaldehyde (PFA), washed with phosphate-buffered saline, decalcified by ethylenediaminetetraacetic acid (EDTA), and embedded in paraffin wax, after which 7-μm frontal sections were prepared for immunostaining and histological analysis. The histomorphological analyses were performed using H&E and Masson’s trichrome (MTC) staining as described previously (Jung et al., 2017). For immunostaining, primary antibodies against TNF-α (cat# ab9739, Abcam, Cambridge, MA, United States); myeloperoxidase (MPO) (cat# bs-4943R, Bioss, Woburn, MA, United States); NESTIN (cat# ab11306, Abcam, Cambridge, MA, United States), and TGF-β (cat# ab92486, Abcam, Cambridge, MA, United States) were used. The secondary antibodies used were biotinylated anti-rabbit or anti-mouse immunoglobulin G (IgG). The primary antibody binding to the fragment was visualized using the diaminobenzidine tetrahydrochloride (DAB) reagent kit (cat# C09-12, GBI Labs, Bothell, WA, United States).

### Real-Time PCR

After 14 and 21 days of hDPSCs cultured with apigenin, total RNA was isolated from cells and complementary DNA (cDNA) was synthesized. Then, a real-time PCR assay was performed to quantify the gene expression with Power SYBR™ Green PCR Master Mix (cat# 4367659, Thermo Fisher Scientific, Waltham, MA, United States). The expressions of genes encoding ALP, β-CATENIN, BMP2, BMP4, BMP7, dentin sialophosphoprotein (DSPP), glyceraldehyde 3-phosphate dehydrogenase (GAPDH), osteocalcin (OCN), and RUNX2 were examined. Each sample was analyzed in triplicates. The results of the assays were normalized to the levels of an endogenous control gene (GAPDH). The primers used in this study are listed in Supplementary Table 1.

### Micro-CT Evaluations

The maxilla after 6 weeks of treatment with apigenin was analyzed using micro-CT imaging (Skyscan 1272; Bruker, Kontich, Belgium), and the three-dimensional (3D) reconstructions were constructed using NRecon software for quantifying the volume of hard tissue formed as described previously (Jung et al., 2017).

### Photography and Image Analysis

All histological and immunostaining slides were photographed using a DM2500 microscope (Leica, Wetzlar, Germany) and a digital CCD camera (DF310 FX, Leica, Wetzlar, Germany).

### Statistical Analysis

The statistical data are expressed as mean ± SD. Comparisons were made between the experimental and control groups using Student’s *t*-test, and *p*-values < 0.05 were considered significant.

## Results

### Human Dental Pulp Stem Cells and Apigenin Treatment

The DPSCs or stem cells from the human exfoliated deciduous tooth (SHED) have important applications in regenerative therapies such as dentin regeneration (Potdar, 2015). In this study, hDPSCs were used as *in vitro* model to examine the effect of apigenin during pulp and dentin regeneration. To examine the cell viability and cytotoxicity, we employed an MTS assay to analyze the proliferation of hDPSCs after treatment of apigenin with various concentrations (Figure 1B). Our results showed that cellular proliferation was not affected in a range of concentrations between 0.1 μM and 5 μM of apigenin after 24, 48, and 72 h (Figure 1B). After ensuring the cell viability, the ALP activity of hDPSCs with various concentrations of apigenin was examined. The highest ALP activity was observed with 5 μM apigenin treatment at day 21 (*p* < 0.05). In contrast, the ALP activity was almost similar to 0.1, 0.5, 1, and 5 μM apigenin at days 7 and 14 (Figure 1C). Therefore, we selected 5 μM apigenin as a suitable concentration for *in vitro* cell cultivation. ALP is one of the earliest markers of osteoblastic cell differentiation (Wrobel et al., 2016), and therefore, we examined the expression patterns of some BMP- and osteogenesis-related signaling molecules on day 14 prior to higher ALP activity in the hDPSCs. Our results showed that both the Bmp- and the osteogenesis-related signaling molecules (i.e., BMP2, BMP4, BMP7, BSP, RUNX2, and OCN) were significantly upregulated in the apigenin-treated specimens compared to controls after 14 days (Figure 1D and Supplementary Table 2). Similarly, the expressions of ALP, BMP2, BMP4, BSP, OCN, and RUNX2 were also increased in the vehicle control compared to the negative control (Figure 1D and Supplementary Table 2). In addition, we also examined the expression patterns of these signaling molecules on day 21, in which, most of the signaling molecules were upregulated in the apigenin-treated hDPSCs (Figure 1D). Particularly, DSPP, one of the important proteins for the mineralization of tooth dentin (Yamakoshi, 2009), was significantly upregulated in the apigenin-treated hDPSCs (Figure 1D). Based on these *in vitro* examinations, we performed *in vivo* animal experiments using 50 μM apigenin as a suitable concentration, which is 10 times of *in vitro* cell cultivation as in the previous study (Kim et al., 2020).

### Inflammatory Reaction Modulation by Apigenin Treatment

**FIGURE 2:**
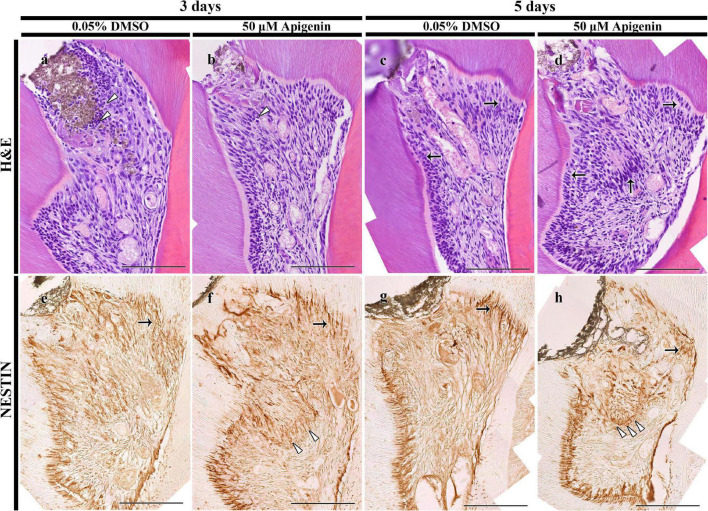
H&E staining and immunolocalization of NESTIN. H&E staining showing more disintegrated cells in the DMSO-control than apigenin-treated pulp cavity after 3 days (arrowheads) **(a,b)**. Increased pulp and odontoblast cells are observed in the apigenin-treated specimen after 5 days (arrows) **(c,d)**. The intense immunolocalization of NESTIN is observed beneath the exposed pulp (arrowheads) and reactionary dentin-forming region (arrows) in the apigenin-treated pulp cavity in both 3- and 5-day specimens **(e–h)**. Scale bars: 50 μm **(a–h)**.

The histological examinations of dentin-pulp tissue were performed using H&E staining as described previously (Jung et al., 2017). After 3 days, more disintegrated cells were detected in the DMSO-control specimen compared with the apigenin-treated specimen (Figures 2a,b). Specifically, the disintegrated cells were observed beneath the exposed pulp in the DMSO-control group compared with the coincided region of the apigenin-treated group (Figures 2a,b). After 5 days, more pulp and odontoblast cells were observed in the apigenin-treated specimen compared with DMSO-control (Figures 2c,d). In addition, the localization of active odontoblast differentiation marker, i.e., NESTIN, was intense in both 3- and 5-day apigenin-treated specimens compared with DMSO-control (Figures 2e–h).

**FIGURE 3:**
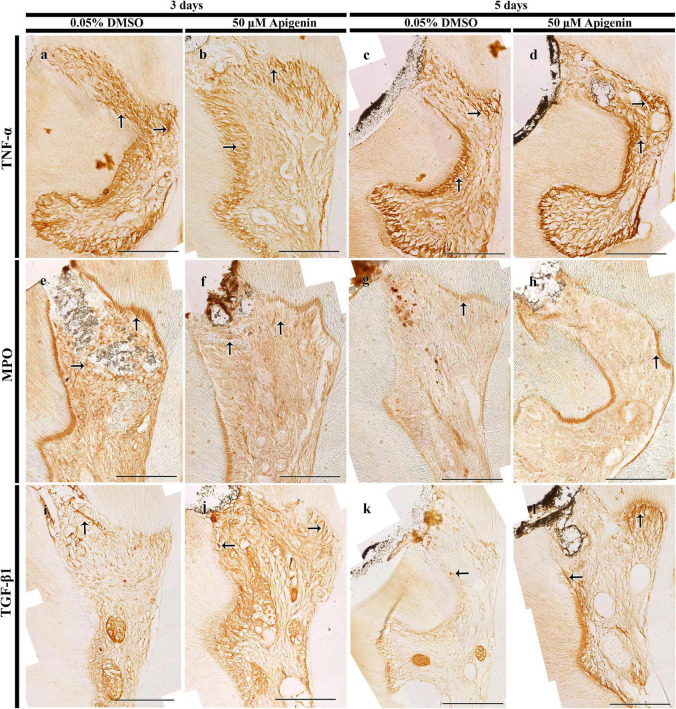
Immunostainings of tumor necrosis factor (TNF)-α, myeloperoxidase (MPO), and transforming growth factor (TGF)-β1. The localization of TNF-α is decreased in both 3- and 5-day apigenin-treated specimens (arrows) when compared to control **(a–d)**. On day 3, the localization of MPO is decreased in the apigenin-treated specimens; however, the immunostaining of MPO is almost similar in both DMSO-control and apigenin-treated specimens (arrows) on day 5 **(e–h)**. In contrast, increased immunostaining of TGF-β1 is observed in both 3- and 5-day apigenin-treated specimens (arrows) when compared to control **(i–l)**. Scale bars: 50 μm **(a–l)**.

The tissues were inflamed after the pulp cavity access preparation, and hence, to examine the role of apigenin in the inflammatory modulation in the exposed and inflamed pulp tissue, immunostainings were performed in 3- and 5-day specimens (Figure 3). The well-known anti-inflammatory markers, i.e., TNF-α and MPO, were examined to understand the apigenin function in exposed pulp inflammation control. After 3 days from pulp exposure, there was a decreased localization pattern of TNF-α in the apigenin-treated specimens (Figures 3a,b). Meanwhile, the intensity of immunostaining of TNF-α was very slightly decreased in the apigenin-treated specimens after 5 days (Figures 3c,d). Similarly, apigenin-treated specimens showed a decreased localization pattern of MPO after 3 days (Figures 3e,f), whereas no obvious differences were observed between the control and the apigenin-treated specimens after 5 days (Figures 3g,h). The *in vitro* experiment showed the upregulation of BMP- and osteogenesis-related signaling molecules after apigenin treatment; therefore, we sought to examine the immunolocalization of TGF-β1 in the *in vivo* animal experiment. Our result showed the stronger positive localization pattern of TGF-β1 in the apigenin-treated specimens compared to control in both 3- and 5-day specimens (Figures 3i–l). Specifically, the intense immunostainings were observed along the reparative and reactionary dentin-forming regions in the apigenin-treated specimens compared with the same region of DMSO-control (Figures 3i–l). Furthermore, the intensities of immunostainings were quantified as weak (+), mild (++), and strong (+++) and prepared as Supplementary Table 3.

### Micro-CT and Dentin-Bridge Evaluations

**FIGURE 4:**
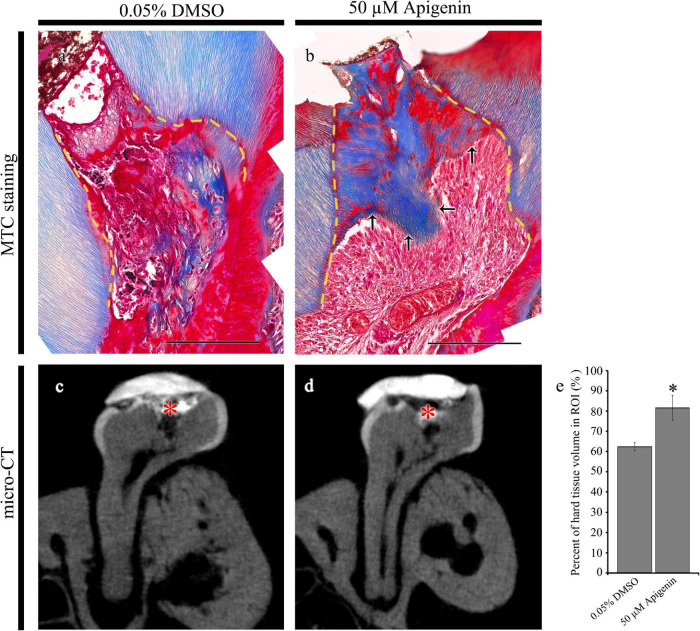
Micro-CT examinations and Masson’s trichrome (MTC) staining after 42 days from cavity preparation. MTC staining showing dentin-bridge (arrows) formation in the apigenin-treated specimen **(a,b)**. Micro-CT showing pulpal access preparation and dentin-bridge formation after 42 days of cavity preparation **(c,d)**. The percentage of hard tissue regenerated within the region of interest after 42 days of cavity preparation (*N* = 3) **(e)**. ROI, region of interest. Red * indicates the region of cavity preparation **(c,d)**. The yellow dotted line indicates the existing dentin in the cavity **(a,b)**. * indicates *p* < 0.05 **(e)**. Scale bars: 50 μm **(a,b)**.

After 42 days of apigenin treatment, MTC staining and micro-CT evaluation were employed to examine the dentin-bridge and the percentage of newly regenerated tissue in the pulp cavity as described previously (Figures 4a–e; Jung et al., 2017). Compared with the DMSO-control, the apigenin-treated specimen showed dentin-bridge formation beneath the exposed area as reparative dentin (Figures 4a,b). The newly formed dentinal tubules were osteodentin-like rather than tubular reparative dentin (Figures 4a,b). Similarly, the percentage of hard tissue volume in the apigenin-treated specimen (81.5 ± 6.1%; *N* = 3) was significantly higher than the DMSO-control (62.4 ± 1.9%; *N* = 3) (Figures 4c–e).

## Discussion

Various procedures, such as tissue-engineered biomolecules and scaffolds are practiced in regenerative endodontics to restore the biological properties of lost tooth structure (Decup et al., 2000; Goldberg et al., 2001, 2009; Dimitrova-Nakov et al., 2014; Njeh et al., 2016; Bottino et al., 2017). In dental caries, the restoration of the biological function of injured pulp and odontoblasts might be one of the plausible ways for tissue regeneration (Lesot et al., 1993). Mostly, the exposed dentin acts as a barrier for preventing further tooth damage; therefore, applying such drugs that facilitate the restoration of the biological function of pulp cells plays a pivotal role in cavity treatment and dentin regeneration (Karakida et al., 2019). Considering these, the local delivery of drug in the exposed pulp using a mouse model system was employed, which we had already established the experimental protocols (Jung et al., 2017). In this study, apigenin, a phytochemical molecule, was introduced into the pulp cavity of the mouse, and its biological role was evaluated through histology, immunohistochemistry, and micro-CT analysis. We selected apigenin, a well-known anti-inflammatory molecule, as one of the drugs for dentin regeneration because the management of inflammatory reaction is one of the key factors during wound healing and tissue regeneration (Eming et al., 2017; Serra et al., 2017).

The inflammatory response, i.e., a complex biological response, would be a hallmark to induce repair response for local tissue recovery (Slavich and Irwin, 2014; Serra et al., 2017), and therefore, the first step of tissue regeneration is the inflammation control, which otherwise can lead to either disease progression or cell death (Hunter, 2012). It is, therefore, the inflammatory and immunological aspects of the pulp cavity should be taken into consideration during cavity treatment (Smith, 2002; Goldberg et al., 2008). The neutrophils and macrophages are the primary innate cells involved in the cytokine pathway, and during tissue repair and the physiological wound healing process, the inflammation subsides after infectious agents are eliminated from the site of injury (Oishi and Manabe, 2018; Goodman et al., 2019). The decreased localizations of TNF-α and MPO in the apigenin-treated specimens suggested that apigenin would modulate early inflammation in the exposed pulp (Figure 3; Goodman et al., 2019). It is proposed that anti-TNF-α therapies are a major treatment of inflammatory diseases (Parameswaran and Patial, 2010), and reducing inflammatory cytokines, such as TNF-α, is the first step for proper tissue regeneration (Goodman et al., 2019). In this study, the reduced level of TNF-α localization in a 3-day apigenin-treated specimen suggests its role in inflammation control toward the tissue repair process (Figure 3), coincided with previous findings (Ginwala et al., 2019; Zhou et al., 2019). In addition, the controlled release of MPO from the infective site indicates the progress of tissue recovery (Khan et al., 2018); however, the prolonged MPO production is not good for tissue recovery (Lee et al., 1995). The decreased localization of MPO in the apigenin-treated specimen suggests that the injured pulp and odontoblast cells subside their inflammation after 3 days; however, after 5 days, the inflammatory reaction recedes and the stage of tissue repair progress (Figure 3; Eming et al., 2017; Serra et al., 2017). Furthermore, various plant-derived compounds inhibit inflammation by reducing cytokine levels (Fürst and Zündorf, 2014), and apigenin, one of the natural flavonoids, also showed early inflammation control in the pulp cavity in this study.

After the pulp was exposed to the oral cavity, there was extensive cell death, and the repair response initiated surviving pulp cells rather than injured odontoblasts. Therefore, the exposed pulp response triggers pulp cells to secrete dentin, which has a bone-like characteristic, called osteodentin; however, if the injured odontoblasts secrete dentin, it has a tubular structure (Ricucci et al., 2014). In this study, apigenin showed elevated osteoblast differentiation-related genes such as RUNX2 and OCN, as well as the ALP activity as in the previous report (Jung, 2014). Apigenin is reported to be involved in regulating different signaling cascades (Ozbey et al., 2019). In this study, the upregulation of BMPs and osteogenesis-related genes in the apigenin-treated specimen showed its modulating roles in TGF signaling, especially during dentinogenesis (Figures 1, 3). Several studies emphasized the importance of cross-talk between BMP and Wnt-/β-catenin during dentin formation and pulp repair (Handrigan and Richman, 2010; Silvério et al., 2012; Yang et al., 2015); however, apigenin showed an inhibitory effect in the Wnt/β-catenin pathway (Ozbey et al., 2019). Interestingly, our result showed that apigenin-treated hDPSCs did not show any changes in the expression of Wnt/β-catenin (Figure 1). These results suggest that apigenin modulates BMP/TGF-β signaling rather than Wnt-/β-catenin signaling during pulp repair and regeneration. The dentin-bridge thus formed would be osteodentin (Figure 4) rather than true dentin formed by the cross-talk between BMP and Wnt-/β-catenin signaling. In addition, the high ALP activity indicates the osteoblast differentiation in the apigenin-treated hDPSCs. Therefore, this study suggests that to recover the local tissue during pulp injury, the modulating role of apigenin in the BMP/TGF-β signaling pathway is remarkable (Figures 1, 3; Niwa et al., 2018). Moreover, TGF-β1 regulates transcription and interacts with the major dentin proteins present in odontoblasts and dental pulp (Unterbrink et al., 2002; Niwa et al., 2018) and, therefore, plays a crucial role during tooth development and pulp repair (Nie et al., 2006). In this study, the modulating role of apigenin in BMPs and TGF-β signaling showed that apigenin applied in the exposed pulp would facilitate repair response initiation in the injured pulp and enhance calcified structure production as a dentin-bridge to prevent further tooth loss as in previous reports (Ricucci et al., 2014; Jung et al., 2017). Odontoblasts are directly injured after cavity preparation; however, a decreased number of disintegrated cells and increased NESTIN localization in the apigenin-treated tooth specimens showed that the injured odontoblasts during pulp cavity preparation are reexpressed, especially beneath the exposed pulp (Figure 2). Furthermore, the increased expression of DSPP in the hDPSCs suggests that apigenin modulates maintaining the secretory activity of injured odontoblasts and pulp vitality through the modulation of inflammation and the facilitation of BMP/TGF-β signaling after dental injury (Figure 1).

## Conclusion

The fundamental process for dentin regeneration is the healing of exposed pulp through the modulation of signaling pathways during dentinogenesis (Chmilewsky et al., 2014; Angelova Volponi et al., 2018). The *in vitro* model that we employed in this study ensured the cell toxicity, viability, and osteogenic differentiation with apigenin treatment, while the *in vivo* model showed the modulating role of apigenin in inflammation control and dentin regeneration with sound dentin-bridge formation. Overall, this study showed that apigenin treatment resolves inflammation by regulating inflammatory cytokines and modulates TGF-β and BMP signaling, which finally facilitate the dentin-bridge formation. Therefore, apigenin could be used as a potential therapeutic agent for regenerating exposed pulp due to dental caries and traumatic injury.

## Data Availability Statement

The original contributions presented in the study are included in the article/Supplementary Material, further inquiries can be directed to the corresponding authors.

## Ethics Statement

The animal study was reviewed and approved by the KNU-2015-136.

## Author Contributions

YA, C-YY, J-HH, and Ja-YK contributed to conception, design, and data interpretation, and critically revised the manuscript. T-YK, E-SL, SS, EP, Ji-YK, S-YC, HY, W-JS, YL, S-YA, C-HA, and J-KJ contributed to data analysis, interpretation, and critically revised the manuscript. All authors gave final approval and agreed to be accountable for all aspects of the study.

## Conflict of Interest

The authors declare that the research was conducted in the absence of any commercial or financial relationships that could be construed as a potential conflict of interest.

## Publisher’s Note

All claims expressed in this article are solely those of the authors and do not necessarily represent those of their affiliated organizations, or those of the publisher, the editors and the reviewers. Any product that may be evaluated in this article, or claim that may be made by its manufacturer, is not guaranteed or endorsed by the publisher.
