# Supplementary material for: Facilitating Reparative Dentin Formation Using Apigenin Local Delivery in the Exposed Pulp Cavity
Source: Front Physiol. 2021 Dec 10;12:773878. doi: 10.3389/fphys.2021.773878 (PMC8703200; doi:10.3389/fphys.2021.773878)
Supplement: Supplementary file 1 [file Data_Sheet_1.docx]

**Supplementary Table 1.** List of primers used in the study

**Supplementary Table 2.** Raw data of ALP assay (A); qRT-PCR: Day 14 (B) and Day 21 (C).

A

B

C

**Supplementary Table 3.** Evaluation of immunohistochemical staining against NESTIN, TNF-α, MPO and TGF-β1.

|  | Group | NESTIN | TNF-α | MPO | TGF-β1 |
| --- | --- | --- | --- | --- | --- |
| + 3 day | 0.05% DMSO | **+** | **+++** | **++** | **+** |
|  | 50 µM apigenin | **+++** | **+** | **+** | **+++** |
| + 5day | 0.05% DMSO | **++** | **+++** | **+** | **+** |
|  | 50 µM apigenin | **+++** | **++** | **+** | **+++** |

+: weak, ++: mild, +++: strong
